# Supplementary material for: Topoisomerase VI senses and exploits both DNA crossings and bends to facilitate strand passage
Source: eLife. 2018 Mar 29;7:e31724. doi: 10.7554/eLife.31724 (PMC5922973; doi:10.7554/eLife.31724)
Supplement: Figure 1—source data 1. — Sequences of oligomers used for biophysical assays presented in Figures 1, 5, 6 and 7. [file elife-31724-fig1-data1.docx]

### Figure 1—Source Data 1. Oligonucleotides used for fluorescence anisotropy and FRET experiments

Sequences of oligomers used for biophysical assays presented in **Figures 1, 5, 6,** and **7**.

| Substrate;  use | Oligonucleotide sequence |
| --- | --- |
| 20 bp duplex; binding assay | 5’-FAM-CGGAAGAGTATGAGTATTCG-3’  5’- CGAATACTCATACTCTTCCG-3’ |
| 30 bp duplex; binding assay | 5’-FAM-CAAAAAGGAAGAGTATGAGTATTCAACATC-3’  5’ GATGTTGAATACTCATACTCTTCCTTTTTG-3’ |
| 40 bp duplex; binding assay, cleavage assay | 5’-FAM- CTATTGAAAAAGGAAGAGTATGAGTATTCAACATTTCCGC-3’  5’-GCGGAAATGTTGAATACTCATACTCTTCCTTTTTCAATAG-3’ |
| 60 bp duplex; binding assay, cleavage assay | 5'‑FAM‑CGCTTCAATAATATTGAAAAAGGAAGAGTATGAGTATTCAACATTTCCGTGTCGCCCTTC-3’  5’- GAAGGGCGACACGGAAATGTTGAATACTCATACTCTTCCTTTTTCAATATTATTGAAGCG-3’ |
| 70 bp duplex; binding assay, cleavage assay  competition assay | 5’ FAM-CCAAATGCTTCAATAATATTGAAAAAGGAAGAGTATGAGTATTCAACATTTCC GTGTCGCCCTTATTCCC-3’  5’‑GGGAATAAGGGCGACACGGAAATGTTGAATACTCATACTCTTCCTTTTTCAATATTATTGAAGCATTTGG-3’ |
| 70 bp duplex; FRET bending assay | 5’ Cy5-CCAAATGCTTCAATAATATTGAAAAAGGAAGAGTATGAGTATTCAACATTTCC GTGTCGCCCTTATTCCC-3’  5’Cy5.5‑GGGAATAAGGGCGACACGGAAATGTTGAATACTCATACTCTTCCTTTTTCAATATTATTGAAGCATTTGG-3’ |
| 16 bp by 20 bp stacked junction; binding assay | 5’-CGAATACTCAGCTCAACCGAAAGGTTGAGCCTTCGCTCGAAAGAGCGAAGTACTCTTCCG-3’  Annealed to oligomer 1 in the 20 bp duplex substrate. |
