# Supplementary material for: Topoisomerase VI senses and exploits both DNA crossings and bends to facilitate strand passage
Source: eLife. 2018 Mar 29;7:e31724. doi: 10.7554/eLife.31724 (PMC5922973; doi:10.7554/eLife.31724)
Supplement: Figure 1—source data 2. [file elife-31724-fig1-data2.docx]

### Figure 1—Source Data 2. Binding affinities of topo VI for different length duplexes.

|  | Duplex length | | | | |
| --- | --- | --- | --- | --- | --- |
|  | 20 bp | 30 bp | 40 bp | 60 bp | 70 bp |
| K_d, app_ (nM) | 427±17 | 84±6 | 49 ±3 | 38±2 | 62±4 |

*Standard errors in fit parameters are reported.
