# Supplementary material for: Topoisomerase VI senses and exploits both DNA crossings and bends to facilitate strand passage
Source: eLife. 2018 Mar 29;7:e31724. doi: 10.7554/eLife.31724 (PMC5922973; doi:10.7554/eLife.31724)
Supplement: Figure 2—source data 1. [file elife-31724-fig2-data1.docx]

### Figure 2—Source Data 1. Apparent kinetic parameters for ATP hydrolysis by topo VI.

| Parameter | Experimental condition |
| --- | --- |

|  | Apoenzyme (basal) | 400 μM bp  sheared salmon-sperm DNA (800:1 basepairs:enzyme) | 400 μM bp  2.9 kb supercoiled plasmid DNA (800:1 basepairs:enzyme) |
| --- | --- | --- | --- |
| k_cat,app_ (ATP/enzyme/min) | N/A* | 1.04±0.09 | 2.63±0.10 |
| K_m,app_(mM ATP) | N/A* | 0.45±0.13 | 0.22±0.03 |
| (k_cat_/K_m_)_app_ (mM^-1^min^-1^) | N/A* | 2.3±0.7 | 11.9±1.9 |
| Relative efficiency |  | ~1x | ~5x |

* data not fit to model due to negligible ATP hydrolysis rates above background.

**Standard errors in fit parameters are reported.

***The ATPase data collected in this particular study cannot distinguish between true and apparent Michaelis-Menten modes. The fitting parameters are therefore apparent in nature, and should not be taken to imply that the ATPase mechanism has been definitively determined.
