# Supplementary material for: Topoisomerase VI senses and exploits both DNA crossings and bends to facilitate strand passage
Source: eLife. 2018 Mar 29;7:e31724. doi: 10.7554/eLife.31724 (PMC5922973; doi:10.7554/eLife.31724)
Supplement: Figure 4—source data 1. [file elife-31724-fig4-data1.docx]

### Figure 4—Source Data 1. Kinetic parameters for DNA dependent stimulation of topo VI ATPase activity.

| Parameter | Topo VI construct | | | | | | | | | | | | | | | |
| --- | --- | --- | --- | --- | --- | --- | --- | --- | --- | --- | --- | --- | --- | --- | --- | --- |
|  | wildtype | | KGRR^AAA^ | | KGRR^EEE^ | | Stalk/  WKxY^AAA^ | | Stalk/  WKxY^EEE^ | | | | H2TH^AAA^ | | | H2TH^EEE^ |
| Sheared salmon-sperm DNA | | | | | | | | | | | | | | | | |
| k_cat-stim,DNA_ (*ATP/enzyme/min*) | 1.11±0.03 | N/A* | | | N/A* | | | N/A* | | N/A* | | 1.11±0.03 | | | 1.03±0.03 | |
| K_stim,DNA_ (*μM bp DNA*) | 61.2±6.3 | N/A* | | | N/A* | | | N/A* | | N/A* | | 56.2±6.1 | | | 60.2±6.7 | |
| Supercoiled plasmid DNA |  | | |  | |  | | | | |  | | |  | | |
| k_cat-stim,DNA_ (*ATP/enzyme/min*) | 2.93±0.14 | 7.40±0.11 | | | 5.60±0.11 | | | N/A* | | N/A* | | 2.76±0.07 | | | 2.30±0.05 | |
| K_stim,DNA_ (*μM bp DNA*) | 56.8±9.9 | 142.6±6.0 | | | 168.1±9.4 | | | N/A* | | N/A* | | 22.3±2.4 | | | 13.2±1.5 | |

*****data not fit to model due to negligible ATP hydrolysis above basal rate.

****** K_stim,DNA_ denotes the basepair concentration (µM) of DNA where half-maximal ATP hydrolysis is attained in the presence of 2 mM ATP.

***k_cat-stim,DNA_ denotes the maximal stimulated ATP hydrolysis rate in the presence of the specified DNA substrate and 2 mM ATP.

****Standard errors in fit parameters are reported.
