# Supplementary material for: Topoisomerase VI senses and exploits both DNA crossings and bends to facilitate strand passage
Source: eLife. 2018 Mar 29;7:e31724. doi: 10.7554/eLife.31724 (PMC5922973; doi:10.7554/eLife.31724)
Supplement: Figure 5—source data 1. [file elife-31724-fig5-data1.docx]

### Figure 5—Source Data 1. Binding affinities of Top6B mutants for different length duplexes.

| Enzyme construct | Duplex length | | | |  |
| --- | --- | --- | --- | --- | --- |
|  | 30bp | 40bp | 60bp | 70bp | |
| Wildtype  K_d,app_(nM) | 84±6 | 49 ±3 | 38±2 | 62±4 | |
| KGRR^AAA^  K_d,app_(nM) | 65±4 | 67 ±4 | 99±2 | 124±6 | |
| KGRR^EEE^  K_d,app_(nM) | 82±7 | 75 ±4 | 119±3 | 193±4 | |
| Stalk/WKxY^AAA^  K_d,app_(nM) | N/A^*^ | N/A^*^ | N/A^*^ | N/A^*^ | |
| Stalk/WKxY^EEE^  K_d,app_(nM) | N/A^*^ | N/A^*^ | N/A^*^ | N/A^*^ | |
| H2TH^AAA^  K_d,app_(nM) | 94±4 | 58 ±3 | 167±9 | 180±12 | |
| H2TH^EEE^  K_d,app_(nM) | 94±5 | 45 ±2 | 109±3 | 103±3 | |
| h** | 1.3±0.1 | 1.6±0.1 | 1.8±0.1 | 1.7±0.1 | |

*K_d,app_ not determined due to inconsistent or poorly defined ΔFA_max_ values compared to wildtype.

**Hill coefficient fit for data better represented by a cooperative binding model.

***Standard errors in fit parameters are reported.
