# Supplementary material for: Topoisomerase VI senses and exploits both DNA crossings and bends to facilitate strand passage
Source: eLife. 2018 Mar 29;7:e31724. doi: 10.7554/eLife.31724 (PMC5922973; doi:10.7554/eLife.31724)
Supplement: Figure 5—source data 2. [file elife-31724-fig5-data2.docx]

### Figure 5—Source Data 2. Affinities of H2TH and KGRR mutants for supercoiled and sheared salmon-sperm DNA as compared to wildtype presented in Figure 1.

| Substrate | Enzyme construct | | | | |  |
| --- | --- | --- | --- | --- | --- | --- |
|  | wildtype | KGRR^AAA^ | KGRR^EEE^ | H2TH^AAA^ | H2TH^EEE^ | |
| K_I,app_ of Supercoiled DNA (nM) | 0.6±0.3 | 7.2 ±1.6 | 14.7±1.3 | 1.3±1.1 | 10.0±0.9 | |
| K_I,app_ of sheared salmon-sperm DNA (nM) | 39.3±2.6 | 161.7 ±11 | 181.7±10.0 | 54.4±13.1 | 52.1±2.8 | |
| Preference for supercoiled DNA | ~60x | ~20x | ~12x | ~40x | ~5x | |

*Standard errors in fit parameters are reported.
