# Supplementary material for: Topoisomerase VI senses and exploits both DNA crossings and bends to facilitate strand passage
Source: eLife. 2018 Mar 29;7:e31724. doi: 10.7554/eLife.31724 (PMC5922973; doi:10.7554/eLife.31724)
Supplement: Figure 6—source data 1. [file elife-31724-fig6-data1.docx]

### Figure 6—Source Data 1. Affinities of wildtype, H2TH and KGRR mutants for stacked junction DNA.

| Substrate | Enzyme construct | | | | |  |
| --- | --- | --- | --- | --- | --- | --- |
|  | wildtype | KGRR^AAA^ | KGRR^EEE^ | H2TH^AAA^ | H2TH^EEE^ | |
| 20 bp duplex, K_d,app_ (nM) | 427±16 | 338 ±11 | 338±15 | 505±22 | 222±11 | |
| 16 x 20 bp junction, K_d,app_ (nM) | 122±4 | 758 ±28* | 1455±52* | 181±8 | 90±4 | |

*K_d,app_ ambiguous due to poorly defined ΔFA_max_.

**Standard errors in fit parameters are reported.
